# What can science and technology studies learn from art and design? Reflections on ‘Synthetic Aesthetics’

**DOI:** 10.1177/0306312716678488

**Published:** 2016-12-27

**Authors:** Jane Calvert, Pablo Schyfter

**Affiliations:** Science, Technology and Innovation Studies, The University of Edinburgh, Edinburgh, UK

**Keywords:** art, collaboration, critical design, critique, design, interdisciplinarity, opening up, synthetic biology

## Abstract

In this paper we reflect on a project called ‘Synthetic Aesthetics’, which brought together synthetic biologists with artists and designers in paired exchanges. We – the STS researchers on the project – were quickly struck by the similarities between our objectives and those of the artists and designers. We shared interests in forging new collaborations with synthetic biologists, ‘opening up’ the science by exploring implicit assumptions, and interrogating dominant research agendas. But there were also differences between us, the most important being that the artists and designers made tangible artefacts, which had an immediacy and an ability to travel, and which seemed to allow different types of discussions from those initiated by our academic texts. The artists and designers also appeared to have the freedom to be more playful, challenging and perhaps subversive in their interactions with synthetic biology. In this paper we reflect on what we learned from working with the artists and designers on the project, and we argue that engaging more closely with art and design can enrich STS work by enabling an emergent form of critique.

## Introduction

In 2009 we embarked on a project called ‘Synthetic Aesthetics’, which paired synthetic biologists with artists and designers. As the social scientists on the project, we initially assumed we would base our analysis on observation of the pairs and their collaborations, but we soon found that we had much in common with the artists and designers. In different ways, we seemed to be trying to do similar things. This led us to the question that motivates this paper: what can STS learn from art and design?

We focus here on the work of three pairs of Synthetic Aesthetics participants, who explored topics as diverse as futility and time, living machines and evolution, and our coexistence with our bacterial symbionts. We found that this work had notable similarities with STS in terms of two shared objectives: ‘opening up’ and ‘critique’. There were also differences, of course, primarily because the artists and designers on the project made tangible artefacts, while we did not (although we recognize the recent interest in making in STS, a topic we address below). Some also aspired to get their hands ‘wet’ and use scientific tools and materials in their work. Another difference is that play, humour and irony was central to their work, but not to ours. We raise the (slightly uncomfortable) question of whether artists and designers are more successful in their engagements with synthetic biology than we are.

To address the question of the paper, we argue that as STS researchers we can learn from the artists and designers on the Synthetic Aesthetics project in several ways. We can learn from their acknowledgement of the extent to which they become implicated in scientific research agendas. Looking at how they negotiate their relationships of obligation can help us reflect on our own. We also think there are opportunities for learning that could result from the bringing together of critical design and approaches to the upstream governance of technologies (such as Constructive Technology Assessment and Responsible Research and Innovation). We are wary, however, of instrumentalizing art and design for STS purposes. Drawing inspiration from work on interactions between social science and design (particularly Michael, 2012a, 2012b), and on interdisciplinary entanglements (e.g. Fitzgerald and Callard, 2015), we argue that one of the most valuable things we can learn is to embrace the experimental and unexpected nature of collaborations with artists and designers. On these grounds, we argue that working with artists and designers can enrich social scientific work by helping us develop an emergent form of critique, where the outcomes are not obvious from the outset, but emerge from the process of collaboration itself.

## Synthetic Aesthetics

The Synthetic Aesthetics project had its origin in a ‘sandpit’: an intensive, competitive, week-long residential grant-writing event called ‘New Directions in Synthetic Biology’, held just outside Washington D.C. in 2009. Synthetic biology, which can roughly (and controversially) be defined as the application of engineering principles to living systems (see Endy, 2005), was at this time a nascent discipline that was rapidly rising in importance in the global funding system.

Sandpits require participants to develop cross-disciplinary research projects over the course of the week. These projects are subjected to real-time peer review, and successful projects are selected on the final day. Five projects were funded from the $10 million made available by the US’s National Science Foundation and the UK’s Engineering and Physical Sciences Research Council. Four of them were science projects and the fifth, Synthetic Aesthetics, aimed to bring together the synthetic biology and art and design communities in ways that were mutually transformative, by initiating paired exchanges between these two groups. The team that emerged to lead the project was made up of two synthetic biologists (Drew Endy and Alistair Elfick), two STS researchers (the authors of this paper) and one designer (Daisy Ginsberg).

The origin of the Synthetic Aesthetics project in a ‘sandpit’ meant that it was somewhat unconventional from the start (one of the ways in which we secured funding was by performing a dance based on the myth of the Golem). The project escaped many of the demands placed on research funded through more conventional mechanisms, and it felt refreshingly open-ended and unconstrained. Another distinctive feature was that it was not conceived of as a social science research project, but as a collaboration between three different disciplinary groups.

In early 2010 the project team advertised for participants and we received over 200 applications from writers, dancers, artists, designers and architects, as well as a range of scientists and engineers. From these we selected six artists and designers and six synthetic biologists, and paired them according to interests and expertise.^1^ We tasked the pairs with investigating design and synthetic biology, with the explicit freedom to take their work in any direction they chose. The artists and designers spent two weeks in the science laboratory, but, rather more unusually, the exchanges were reciprocal, so the scientists and engineers spent an equal amount of time in the art studio. We refer to these paired reciprocal exchanges as the ‘residencies’ below.

## Methodological preliminaries

At the start of the project, we, the STS researchers on the project, planned (perhaps naïvely) simply to observe and document the interactions between the synthetic biologists and the artists and designers, and to interview all the participants in a methodologically conventional manner. But once we started work, we were immediately struck by the similarities between our objectives and those of the artists and designers. Although they were a diverse group, they all, like us, wanted to forge new collaborations with synthetic biology. The three on whom we focus in this paper also aimed, as we did, to critically interrogate the science by provoking reflection about its social and political complexity, explore implicit assumptions and possible alternatives to these, and question the dominant futures imagined by the scientists and engineers.

Because of these unexpected similarities, our work in the project became a shared investigation into the nature of synthetic biology, using the tools of STS and art and design, rather than an investigation into the collaborations between scientists and artists. This reflects on going anthropological movements towards studying *with* people instead of making studies *of* them (Ingold, 2013), and from thinking in terms of ‘epistemic partners’ rather than ‘informants’ (Holmes and Marcus, 2008).

More practically, once we started our fieldwork we realized that the way we had set up the project challenged any routine methodological positioning of ourselves as ethnographic observers. In the exchanges, we had inadvertently created a situation in which the scientist or engineer in their laboratory was being followed by their paired artist or designer, both of whom were being followed by a social scientist (one of the authors of this paper). All of us, in turn, were being followed by Daisy Ginsberg, who was taking photos, filming interactions, and asking challenging questions. As a critical designer with an interest in ‘guerrilla tactics’, she liked to joke that one of her aims was to subvert the social scientific ethnography. This set-up of three new people descending on a science lab was repeated in all the other residences. The situation was reversed in the art studio, where the scientist/engineer became one of the ‘followers’. This meant that difficult questions arose about who was studying whom. In a sense, we were all studying each other.

Despite our shift in methodological focus towards a shared endeavour, we persisted with our planned interviews and ethnography. We observed and documented the six four-week exchanges and conducted semi-structured interviews with each of the 12 residents on at least two different occasions. Finally, at the end of the project the team wrote a book intended for a broad audience (Ginsberg et al., 2014) in which the art/science pairs co-authored chapters on their joint work. These chapters were a further exercise in collaboration and provide data on which we draw here.

## Reference points

There is a great deal of literature on art/science collaboration (e.g. Dixon et al., 2011; Kac, 2006; Stracey, 2009), but this is not our primary focus here. We are interested in the similarities and differences between STS and art/design in collaborations with scientists and engineers, rather than art-science interactions *per se*. In this way, we are building on recent work that has started to explore the complementarities between social science and art and design, particularly Michael (2012a, 2012b), Horst and Michael (2011) and Horst (2011), as well as Barry et al. (2008) and Born and Barry (2010), who outline three ‘logics of interdisciplinarity’ that inform our analysis.

These three logics provide different rationales for interdisciplinarity: to help scientific research become more accountable to society (the logic of accountability); to facilitate the contribution of scientific research to economic growth (the logic of innovation); and to produce ‘new objects and practices of knowledge’ (Barry et al., 2008: 42) through interdisciplinary research (the logic of ontology). According to the logic of ontology, something that would not have happened otherwise comes about through the collaboration. As Born and Barry (2010) explain (in relation to art/science interdisciplinarity): ‘science is understood not as self-sufficient or complete, but as transformed and enhanced through its engagement with art, just as art is transformed and enhanced through engagement with science’ (p. 105). The logic of ontology is, for us, the most interesting and powerful form of interdisciplinarity, and we return to it below.

## Critical design

Alongside these academic reference points, it is also necessary to introduce the field of critical design. This is, of course, just one approach to contemporary design, but we focus on it here because Daisy Ginsberg, the design fellow on the project, was very influential in its direction and was trained as a critical designer by Antony Dunne and Fiona Raby, widely regarded as leading figures in the field.

Critical designers distinguish their work from commercial design because they use design as a tool to provoke reflection about the social, political and economic complexity of technology. They produce speculative objects that make abstract ideas tangible and open to discussion. They argue that this can ‘help us see that the way things are now is just one possibility and not necessarily the best one’ (Dunne and Raby, 2013: 66). Rather than making predictions, critical designers explore possible futures using design as a means of investigation (Ginsberg, 2014).

We can think about critical design in the light of Latour’s discussion of designed artefacts. Latour shows how design helps us see that what we might have thought were ‘objects’ or ‘facts’ are actually ‘things’ or ‘matters of concern’. He argues that design brings to the fore the ‘disputed assemblages’ (Latour, 2009: 6) that constitute artefacts, but are often overlooked. Design can demonstrate that ‘many participants are gathered in a thing to make it exist and to maintain its existence’ (Latour, 2004: 246). To put it in Latour’s terms, it is almost as if critical designers set out to create ‘matters of concern’. They often refer to their work as ‘design for debate’ (Dunne, 2008), and they aim to create things around which we can have a discussion, around which we can gather.

It is helpful to give an example of a critical design artefact, and we have chosen one that was important to the Synthetic Aesthetics project, because it led us to recruit Daisy Ginsberg into the team. In 2009, Daisy and her colleague James King worked with a group of undergraduate synthetic biologists at the University of Cambridge who engineered the bacterium *E. coli* to express a range of colours that are visible to the naked eye. They thought this application could potentially be useful in detecting levels of pollution and producing an output that was easily readable. Daisy and James speculated about other possible uses of this technology,^2^ and they imagined a future (circa 2039) specially-designed probiotic drink that interacts with human gut bacteria to produce an easily visible output reflecting one’s disease state – coloured faeces. They produced a suitcase of (fake) excrement, and took it across the Atlantic to the International Genetically Engineered Machine (iGEM) competition at the Massachusetts Institute of Technology. They had not paid the fee to present at the conference, so they gave guerrilla-style presentations, taking small groups of conference-goers (including one of us) aside in the breaks and presenting their design future. The climax of each of these mini-presentations involved opening the suitcase to reveal the coloured turds – the imagined future designed output.

This ‘Scatalog’ challenges taboos, particularly because the excrement it imagines is something that is visceral and smelly and very different from the image of synthetic biology that is usually projected, in which we either see images of shiny, clean laboratories or equally shiny double helices. Since 2009, the Scatalog has become (in)famous in the synthetic biology community. In fact, at a plenary session at a synthetic biology conference in 2011, scientists from the Beijing Genomics Institute used it in their presentation to represent the desired end-point of their ongoing research. (It was rather unsettling for the critical designers to see what they thought of as an ironic intervention being embraced by the international scientific establishment.)

## The project

With these preliminaries in place, we now turn to Synthetic Aesthetics project itself, and its reciprocal exchanges between scientists and engineers and artists and designers. All six pairs produced thought-provoking work, but for reasons of space we reluctantly leave out discussion of those collaborations involving a composer, an architect, and a product designer (see Ginsberg et al., 2014). We give brief snap shots of the remaining three projects, which we expand on below in our discussion of the similarities and differences between their work and ours.

### Oron and Hideo

The first pair is Hideo Iwasaki, a microbiologist from Waseda University in Tokyo, and Oron Catts, a bioartist who has built his own unique institutional niche – a lab/studio space called Symbiotica – at the University of Western Australia. Hideo works on cyanobacteria, which are photosynthetic bacteria that have circadian rhythms, and the pair quickly decided to use cyanobacteria in their collaborative work. Cyanobacteria are potentially useful in many applications including bioremediation, so the pair started by discussing the idea of engineering cyanobacteria to digest the silicon in discarded computer chips. One of the products of this breakdown would be gold, but they realized that another would be ‘fool’s gold’, which looks like gold but is widely regarded as a useless substance. They explored the idea of engineering the metabolic pathway so it would preferentially produce fool’s gold. This provoked them to ask what would happen if synthetic biology was directed towards the production of useless products. This is a particularly pertinent and destabilizing question in a field that is heavily oriented around applications and utility.

After further discussion, the pair decided to focus their work on the topic of time, as manifested by cyanobacteria. They were particularly interested in exploring the links between biological and geological time, the sweep of which extends from the beginning to the end of the Earth. They started from the observation that cyanobacteria operate at many different timescales. They have rapid metabolic processes, and exhibit slower daily rhythms in their circadian cycles. But they also act on a much longer timescale because they were the organisms that first converted the Earth’s atmosphere to oxygen and made it habitable for life as we know it. The early Earth was covered in ‘living rocks’, clumps of minerals deposited by photosynthesizing cyanobacteria. Similar rocks can be found in the bleak and beautiful saline lakes of Western Australia, where the pair carried out a stretch of their collaborative work.

One of the aims of Oron and Hideo’s project was to introduce humility by challenging us to look at all human activities, including synthetic biology, from the perspective of geological time. As they put it in their jointly-written chapter: ‘[i]n a few million years, the human era may be just another thin layer in the rock formation, a humbling thought’ (Catts and Iwasaki, 2014: 187). This perspective is not one we normally adopt when we think about emerging technologies, so their work encourages a radical shift in our present-oriented perception of synthetic biology.

### Sascha and Sheref

The next collaboration involved a protocell scientist, Sheref Mansy, the head of a laboratory in the green and mountainous region of Trento, Italy. He was willing to combine the demands of running a lab with collaborating with his paired artist, Sascha Pohflepp. Sheref’s research involves the creation of extremely simple living systems from non-biological components. He is interested in finding the absolute minimum that is needed for something to be alive. Sheref’s research focus led the pair to explore questions about the defining features of life and they developed a shared interest in evolvability as a key feature that distinguishes living from manufactured systems.

The second leg of their exchange took place at Mediamatic, an art and technology studio in Amsterdam, and a very different environment from Sheref’s lab in Italy. (‘I’ve just told my lab I’m doing crazy shit for a couple of weeks’, Sheref explained.) Here the pair decided to focus on the question: ‘Are inanimate machines only an interlude in history?’ They were struck by the fact that we relied on what they termed ‘animate machines’ (horses, oxen etc.) for long periods of our history, and even when non-living machines took over, these were often modelled on living things (for example, horsepower is still the standard of measurement for engine power). They became interested in what kinds of living machines synthetic biology might deliver, and what would happen if these machines evolved. They even explored the idea of an evolving steam engine.

What is notable about this project is that it addresses a contradiction at the heart of synthetic biology. If we take evolution to be a defining characteristic of life, then to be alive a ‘living machine’ must evolve, but if it evolves it may no longer possess the stable, predictable properties we expect of our machines. In their jointly written chapter, Sascha and Sheref argue that synthetic biologists must inevitably ‘share authorship with the innumerable forces that drive Darwinian evolution’ (Mansy and Pohflepp, 2014: 242).

### Sissel and Christina

The final pair was Christina Agapakis, then a PhD student in synthetic biology at Harvard University, and Sissel Tolaas, a smell artist based in Berlin. Sissel’s mantra is ‘nothing stinks, only thinking makes it so’.^3^

In their project they decided to explore our contradictory relationship with bacteria. They became intrigued by the fact that we live in a world filled with microorganisms, but we consider bacteria to be dirty and hazardous and we seek to exterminate them using cleansers and medical technologies. The medium through which they decided to explore these ideas was cheese. This is because some of the bacteria that give cheeses their distinctive smells are also found on human skin, probably due to early artisanal cheese-making practices where people used their bare hands. In their project, Sissel and Christina extracted bacteria from different areas of human skin (including noses, toes and armpits) and used it to make cheese in the laboratory.

This project is playful, but it raises profound questions about the reality of the human microbiome and the relationship between humans and bacteria, pointing to the fact that bacterial and human cultures co-evolve. It also challenges the boundaries between our bodies and the food that we eat, since bacteria do not distinguish between them in the same way as we do.

## Similarities

### Opening up

Turning to the similarities between these three art/design projects and our STS work, one that quickly became evident was our shared desire to ‘open up’ synthetic biology to a broader range of perspectives, and expand existing ways of imagining the field. Stirling (2008) describes ‘opening up’ as drawing attention to the often implicit assumptions that underlie discussions of a technology. He shows how, in policy contexts, opening up can enable new questions to be asked, marginalized perspectives to be included, and alternative technological pathways to be explored. Although the artists and designers were not aware of Stirling’s work, we do think there are important resonances between STS and art and design in this respect. The aspiration to ‘open up’ synthetic biology was a feature of all three of the pairs’ projects.

We have seen how Oron and Hideo confront our narrow conceptions of synthetic biology and its future by adopting the perspective of geological time. But more broadly, much of Oron’s work is motivated by his desire to bring new voices into the discussion of the life sciences and to explore the ambiguities that emerge. In an interview he explained: ‘I think art is in this really privileged position to engage in questioning and opening up areas of exploration as opposed to smoothing over them’ (OC interview Perth). This desire to open up new areas to artistic exploration was one of the main reasons why he wanted to be part of the Synthetic Aesthetics project. He said,

I think there is an urgent need for artists to explore major issues that are being raised by synthetic biology because, if we are not there at the very beginning, it will be too late to be able to show alternative ways in which this knowledge can be applied. (OC interview Perth)

Oron also argued that the ability to open up the discussion of technology was a feature of art rather than design. He elaborated,

Very few professions actually are allowed to spend their time engaging in developing something only for it to be contested. Designers and engineers are trained to find solutions that are going to bring closure in a sense; they’re not interested as much in the idea that what you’re engaging with is designed to be questioned. (OC interview Perth)

In their collaborative work, Oron and Hideo wanted to initiate a critical discussion that did not have a defined endpoint, but that was characterized by continuous exploration.

Sascha and Sheref saw their own interdisciplinary interaction as a form of ‘opening up’. Sheref had applied to be part of the project because he hoped that his interactions with artists and designers would ‘stimulate creativity, different perspectives, different thoughts’ (SM interview Trento). He stressed the importance of creativity to his scientific work and hoped engagement with creative professionals would help him do better science. At the start of the residency he explained what he wanted to get out of the project:

I would love – it’s asking for a lot so if this doesn’t happen then I won’t be disappointed – but I would love if there was a moment in which I thought, ‘ah, I’d never looked at it in that way, but perhaps that’s something that I should think about more’. That would be great. (SM interview Trento)

Sascha, like Oron, drew a distinction between art and design. He explained that he wanted to participate in Synthetic Aesthetics as an artist because design was about finding solutions and ‘problem-solving, improving the world, you know, which is a very noble effort, but it’s more about constraints, whereas art is more about actually opening, posing questions than delivering answers’ (SP interview Trento).

Sissel and Christina engaged in opening up by finding novel artistic uses for established biological tools and techniques. Much of Sissel’s work has followed from being given access to sophisticated technologies from the scent and flavour industry and using them in idiosyncratic ways. She regarded her project with Christina in the same light. Having experienced the synthetic biology laboratory, she was struck by the apparent narrowness of the field. By re-purposing synthetic biological knowledge, she hoped to show the possibilities overlooked by scientists and engineers by exploring new, in this case more playful, directions for their research. Again, it is worth noting that the distinction between art and design was raised in this collaboration. As Sissel noted, the artist can ‘dare to be less practical’ (ST interview Boston).

### Critique

A second similarity we found between our STS work and that of the artists and designers is what we are calling ‘critique’. What is meant by critique is obviously a moot point here, and we return to this issue below, but we start by drawing on our actors’ use of the term.

Oron and Hideo talked about critique more explicitly than any of the other pairs. At one point Oron reflected: ‘I suppose the greatest challenge from my perspective is how to engage critically’ (OC interview Perth). He went on to explain that any engagement with a field like synthetic biology normalizes it, even if that work explicitly aims to be critical, and that by being part of the project there was a danger that he could end up providing a service without much influence.

One form of critique found in the pair’s work is the attention they draw to hubris and control. In their joint chapter they write: ‘there is a need to question the underlying hubris of human intentions to control life. In this context, we hope to demonstrate that time can be used as an instrument for humility’ (Catts and Iwasaki, 2014: 185). These concerns resonate strongly with work by Wynne (1992) and Jasanoff (2003), who argue that it is necessary to acknowledge the limits of prediction and control in science and engineering. Jasanoff (2003) advocates ‘technologies of humility’, which she describes as attempts ‘to come to grips with the ragged fringes of human understanding – the unknown, the uncertain, the ambiguous, and the uncontrollable’ (p. 227).

Sascha and Sheref did not talk about critique as often as Oron and Hideo, but in some ways their project is a critique of the whole synthetic biology agenda, because they point to the contradictions of trying to design ‘living machines’, which is the guiding objective of much synthetic biology. As mentioned above, their central focus was on evolution as a defining feature of life, and their final output, an exploration of the nature of living machines, shares many themes with critical work in STS; their co-authored chapter even cites Lily Kay and Bruno Latour. In this particular case, the boundaries between their contribution and ours become very blurred.

Sissel and Christina did not explicitly discuss critique, but one of the most interesting aspects of their work was that it allowed them to draw critical conclusions about the metaphors and analogies used in synthetic biology. The field makes heavy use of metaphors from engineering, and is saturated with analogies to resistors, capacitors and electronic circuits. By pointing to the interconnectedness and complexity of the bacterial cultures in their cheeses, Sissel and Christina were able to provoke different ways of thinking. They write in their joint chapter: ‘rather than modeling synthetic biology on computer engineering, cheesemaking might be an engineering paradigm that allows for the design, construction, and maintenance of complex living worlds performing incredible feats of metabolism’ (Agapakis and Tolaas, 2014: 282). This is a profound challenge to the way synthetic biology is currently framed.

## Differences

### Making

Moving on to consider the differences between our STS work and that of the artists and designers, one that is superficially obvious is that artists and designers make things that have a materiality and a physicality that our academic contributions usually lack. We recognize that the recent turn to ‘making’ in STS aims ‘to use material forms of engagement with technologies to supplement and extend critical reflection’ (Ratto, 2011: 253), and we see Synthetic Aesthetics as continuous with this endeavour, but the artists and designers in the project prioritized making in a way few social scientists would. For example, in JC’s first conversation with Sascha *en route* to Trento, the question he most wanted to ask of the other residencies was: ‘what are they going to make?’

This emphasis on making appears to be interconnected with ideas about what it is to be an artist or designer. Maeda (2010), for example, says: ‘Being an artist, I feel that art comes from the inexplicable urge to manifest a feeling, intent, or question as a specific, tangible experience.’ Some artists and designers also argue for the unique contribution that making brings to their engagements with science and technology. Dixon (2008) maintains that ‘only a material confrontation can be surprising and astonishing’ (p. 685), and Carey et al. (2014) say that it is because they produce artefacts that designers ‘can present a powerful vision of the future, facilitating visceral and tangible forms of engagement with innovation and possibility’ (p. 176).

The power of a non-verbal output is clearly demonstrated by Sissel and Christina’s project. The cheeses have been catalysts and focal points for discussions around synthetic biology in Berlin cheese shops, synthetic biology conferences, and larger events like the South by Southwest festival in Texas. In each case they have driven discussions that extend far beyond the cheeses themselves to encompass questions about humans’ relationship to bacteria, the complex interplay between science, technology, consumer products and food, and the role that synthetic biology may play in our lives. All these issues are made tangible through the medium of cheese.

The potential for art to provide an alternative, non-verbal, form of expression was particularly important for Oron, who argued that ‘the issues that synthetic biology raises are way beyond science and engineering, so there’s a need to try and culturally articulate stuff that we don’t even have a language to describe’ (OC interview Perth). He maintained that words are not necessarily the best form of expression, and put the point directly (during an interview, tellingly) by saying ‘I’m really interested in doing stuff, not just talking about it!’ (OC interview Perth). This point was also made by Daisy who said that ‘too much self-reflection is a problem’, because it inhibits making.

Oron did point out that one downside of non-verbal work is that there is the danger of ‘misunderstanding or misframing or misusing the artistic work for purposes which are totally opposing the intentions of the artist’ (OC interview Perth). Although written texts can be (and often are) misinterpreted, the danger of this happening with artworks is even greater. For example, the Scatalog appeared in the UK newspaper the *Daily Mail* under the heading ‘Scientists create yoghurt that changes the colour of your poop to diagnose illness’ (Wrenn, 2012), demonstrating the propensity of some groups to believe speculative technological futures.

Because of its ability to provoke thought and discussion, one of the residents described artistic work as: ‘really vivid science studies’ (HI interview Perth), but interestingly, they did not regard us as fellow makers. While they were making we seemed to be merely writing. We do not necessarily accede to this categorization, but we note that making was a way in which the artists and designers in the project contrasted us with their paired synthetic biologists, who similarly put an emphasis on constructing artefacts.

To add an additional twist to their emphasis on making, bioartists such as Oron use the same tools and materials in their work as those used in the laboratory by scientists. In this way, their approach is much more ‘hands-on’ than is most STS work. As Oron explained, what interested him was ‘how we can actually use the very same technology and the very same question of manipulating life forms as a vehicle for expressing those questions’ (OC interview Perth). Kelty (2010) similarly notes how bioart makes use of ‘green fluorescent proteins, petri dishes, tissue culture manuals, and genetics databases’, but ‘configures them in ways that try to provoke, transgress or re-design our understandings of life’ (p. 7). And Sissel and Christina’s collaboration was a clear example of repurposing the tools of science for a different, artistic end.

Both Daisy and Oron were committed to the transformative power of laboratory work. They argued that by getting one’s hands ‘wet’ (or ‘dirty’ perhaps), one becomes implicated in the process of manipulating life. This makes it hard to take a distanced, detached stance; a stance often attributed to us as social scientists, who seemed to spend most of our time on the sidelines, taking notes. There was also an implication that this hands-on involvement allowed for a deeper critique than we could ever accomplish as STS scholars. We return to this issue of being a ‘participant’ rather than a ‘spectator’ below.

### Play and humour

As well as regarding themselves as ‘makers’, the artists and designers also thought of themselves as provocateurs, jesters or saboteurs, again in contrast with us. Of course STS has a tradition of playful work exhibited by authors like Haraway, Ashmore, Mulkay and Woolgar, and we have tried to adopt ‘trickster’ roles ourselves in our interactions with synthetic biologists, with mixed results (see Balmer et al., 2015). But play, humour and irony were central to the work of the artists and designers on the project, as shown by Daisy and James King’s guerrilla tactics at the undergraduate synthetic biology conference described above, Oron and Hideo’s attempt to ‘engineer futility’ into cyanobacteria (Catts and Iwasaki, 2014: 198), and the audacity of armpit cheese.

### More successful?

As we became aware of these similarities and differences between STS and art and design, our work on the project started to become accompanied by a slightly uncomfortable question: are artists and designers more successful in their engagements with synthetic biologists than we are? The answer to this question rests on what is meant by ‘success’, of course, but as a starting point, many synthetic biologists seem to be happier to collaborate with designers than with social scientists, and these collaborations often exhibit less divergence in expectations. This is partly because design is central to engineering, so the value of a designer’s contribution is perhaps easier for synthetic biologists to acknowledge than the more amorphous contribution that a social scientist makes in his or her papers and talks (the former often taking many years to reach publication).

Artists and designers are also often considered to possess skills that the STS researcher is perceived to lack. Sheref, for example, wanted to collaborate with an artist because of the creative input that they would bring; something that is not so readily associated with an STS researcher. There also seemed to be an expectation that artists and designers would be playful, challenging and sometimes subversive in their engagements with synthetic biology, an expectation that is not generally associated with social scientists.

Another measure of the success of the artists and designers is that the tangible artefacts they produce have an immediacy and an ability to travel (Wilkie, 2011), and are more discussed by the synthetic biology community than our social scientific papers. But this raises questions about whether the critical interventions of the artists and designers are more likely to become folded into the mainstream of the field (as we saw with the uptake of the Scatalog by the Beijing Genomics Institute). Does this warmer welcome for designers come at the cost of being taken less seriously? Are artists and designers more likely to be co-opted, assimilated and neutralised than STS researchers are? We return to these questions below.

Since designers and artists can be seen as simultaneously less threatening and more obviously useful in synthetic biology, this raises questions about whether they are in competition with us, filling a space that might otherwise be filled by social scientific enquiry. Some writing by critical designers seems to support this suggestion. For example, Daisy writes that ‘[a]n emerging role for the designer is a form of social critic’, and says her aim is to ‘develop a type of applied, speculative bioethics. Researching science and engaging in discussion with scientists’ (Ginsberg, 2014: 66) – which sounds very much like certain approaches in STS. Similarly, on the back cover of their book *Speculative Everything* we are told that ‘Dunne and Raby continue to inspire and challenge us to consider design as a unique mode of sociocultural inquiry’. Is this encroachment of design onto the ‘sociocultural’ territory something we should fear or embrace?

Perhaps some of the competition we felt was based on the fact that Synthetic Aesthetics was not an art/science project; it was an art/science/social science project. This meant that the interactions were between three disciplinary groups, rather than two – the latter being far more normal in interdisciplinary work. This resulted in unusual group dynamics, constantly shifting allegiances, and a tendency for all of us (artists, designers, synthetic biologists and STS researchers) to attempt to articulate the distinctive contributions of our own (hybrid) disciplinary perspectives.

There was an asymmetry in this threesome, however, because the artists and scientists were funded to spend time in each other’s workspaces and expected to come up with an output that was co-produced. The pairs worked closely together and all produced jointly-authored papers with surprising ease. One reason why their collaborations may have been successful is that the residencies were set up so that they *had* to collaborate. Perhaps this shows how important the starting positions are in a cross-disciplinary collaboration. STS researchers are rarely funded specifically to produce a joint output with a scientist or engineer. And we have never come across a project where a scientist has to spend the same amount of time in an STS department as the STS researcher spends in his or her scientific fieldsite.

## What can we learn?

We now turn to our central question: what can we learn from the artists and designers in the Synthetic Aesthetics project? In raising this question, we are not intending to set up a hierarchy with art and design above STS, or to imply that art and design have nothing to learn from STS. Instead, our aim is to reflect on what were, for us, novel and stimulating interactions, and draw out some features we think might be interesting or useful for other STS researchers.

We realize that asking ‘what can STS learn?’ raises questions about what STS is. We are loath to attempt to make an authoritative statement on this issue, particularly since STS is notoriously heterogeneous and, as we see it, incorporates a spectrum of work from the theoretical to the descriptive, the critical, the normative and the activist (see Rip, 1999; Sismondo, 2008). But it is the case that this interdisciplinary project did make us re-identify with our own field and reflect on its nature – one of the results of our reflection being this paper.

With these caveats in place, we lay out three ways in which we think we can learn from the artists and designers. The first is in terms of the ways in which they negotiate their relationships with scientists and engineers. The second is with respect to our work on the governance of emerging technologies. Finally we reflect on what we might achieve together: an emergent form of critique.

### Being implicated

In both Europe and the US, there are increasing pressures on STS researchers to become tagged onto scientific grants to deal with the ‘ethical, legal and social issues’ (Balmer et al., 2015). STS scholars can become heavily involved in the topics we study and can come to play a role in the development of new scientific fields. The artists and designers in the project were well aware of the extent to which they are implicated in similar ways. This draws attention to what Barry et al. (2008) call the ‘subordination-service mode’ of interdisciplinary work. Both STS researchers and artists and designers working in new technological fields are often expected to perform a service role and facilitate the progress of science to market. Looking at the ways in which artists and designers deal with their complicity can help us reflect on our own.

Oron and Hideo were wary that their collaborative work might become folded into the construction of synthetic biology. In their joint chapter they write: ‘Synthetic biology is a contemporary example of a field that employs artists and designers as part of a concerted effort to engineer public acceptance for a technology that does not yet exist’ (Catts and Iwasaki, 2014: 194). Oron was concerned that the Synthetic Aesthetics project would be interpreted in this way. However, he was also keenly aware that he had chosen to be implicated, because he had chosen to use the tools and materials of synthetic biology to produce art. In an interview, he explained:

The artists who are involved with it are implicated within the whole process; they can’t take a distanced stance, they actually have to engage, they can’t be self-righteous about it. (OC interview Perth)

This raises questions about the tension between distance and critique. In the previous section, we asked whether the greater success artists and designers had in their collaborations with synthetic biologists brought with it the danger of their work being co-opted and assimilated. Although some artists and designers celebrate their closeness to the science, is critique actually stronger from outside, when there is some distance? These are long-standing questions in STS, and reflecting on the ways in which artists and designers are enrolled in synthetic biology throws our own forms of critical engagement into relief.

Artists and designers do adopt a range of strategies to protect their autonomy. For example, by having his own laboratory dedicated to artistic research, Oron is not dependent on a scientific sponsor or collaborator to carry out his bioart. Dunne and Raby (2013) suggest another approach – to ‘work independently with scientists as advisors rather than creative partners’ (p. 54) – on the grounds that in this situation they are freer to set their own agenda. This arrangement was clearly precluded by the set-up of Synthetic Aesthetics, because the work had to be collaborative. The reciprocity of the exchanges meant that the lines of obligation were not straightforwardly unidirectional, however, and the creative process was a shared one. We elaborate on the significance of this point below.

### Upstream governance

We think the second area of cross-learning between STS and art and design is in respect to the upstream governance of emerging technologies. For STS researchers coming across critical design, it is striking how much it has in common with approaches such as Constructive Technology Assessment (Schot and Rip, 1997), Anticipatory Governance (Barben et al., 2008), and more recent formulations in terms of Responsible Research and Innovation (Owen et al., 2013). It is under these headings that STS researchers often contribute to deliberations about technological choices. All of these approaches aim to create novel opportunities for diverse groups to come together in the early stages of the development of a technology. They use tools such as scenarios to elicit alternative visions of the future, which can help articulate a wider range of pathways than would be envisaged otherwise.

In recent discussions of Responsible Research and Innovation, the similarities to critical design are notable. For example, Owen et al. (2013) say that their aim is ‘to explore other pathways to other impacts, to prompt scientists and innovators to ask “what if …”’ (p. 38). Similarly, in their description of their critical design work, Dunne and Raby (2013) explain how it often takes ‘the form of scenarios, often starting with a what-if question’, and that these questions ‘are intended to open up spaces of debate and discussion’ (p. 3). They even say that they want their work to ‘play a role in the democratisation of technological change by widening participation in debates about future technologies’ (Dunne and Raby, 2013: 49). This statement could come straight out of a description of CTA. Because of these similarities, there seems to be rich potential for bringing these areas of investigation together.

There are already openings for this type of work. For example, CTA-informed socio-technical scenarios workshops have started to make use of prototypes (Rip and te Kulve, 2008), and technology assessment has embraced the speculative in the form of ‘vision assessment’ (Grunwald, 2004). A suggestion that emerges from the Synthetic Aesthetics project is that we could build on these approaches to develop methods that harness the skills of artists and designers in their imagination of alternative futures, and their abilities to express things in a tangible form. The physicality of the outputs could broaden discussions in unanticipated ways, leading to novel means of envisioning the future of engineering living things.

This would be a nice neat conclusion of the paper: that we do our social scientific research but introduce an object – a suitcase of poo, for example – to provoke conversations. But because of our unexpected, surprising and sometimes unsettling experiences of working with artists and designers we are not entirely satisfied with this conclusion, since there is a danger of putting art and design into an instrumental role, performing a service for STS. Simply tasking designers with making speculative prototypes for discussion does not embrace the ways in which art and design could be part of an ongoing discussion from which something more unexpected could arise. Despite the emphasis given to making by the artists and designers on the Synthetic Aesthetics project, what we gained most from our collaborations with them were not primarily ‘things’ we could wheel out to stimulate debate, but thought-provoking conversations that introduced ideas and assumptions different from our own.

This resonates with the ‘critical making’ movement discussed earlier, because rather than prioritizing the object, the emphasis here is placed on the process, on ‘the act of shared construction, joint conversation, and reflection’ (Ratto, 2011: 253). And it is precisely the collaborative process that we have come to value most through our involvement in Synthetic Aesthetics. This emphasis encourages us to take up Ingold’s (2013) call to embrace ‘the speculative, experimental and open-ended character of arts practice’ (p. 8), and the possibilities it allows. Like Born and Barry (2010), we think that art/science projects can ‘contribute to the generation of something new within scientific practice itself, challenging the boundaries of disciplinary authority’ (p. 114). Instead of seeing artists and designers primarily as producers of artefacts, we can perhaps make the most out of our collaborations if we start to see them as epistemic partners on an exploratory journey.

### An emergent form of critique

This leads us to our central argument: by working with artists and designers we can develop what we are tentatively calling an emergent form of critique, which could expand our critical capacity and provide alternative entry points into discussions of synthetic biology.

We have been inspired here by Michael’s discussion of the emergent potential of speculative design. Michael (2012a) describes how this approach to design is not concerned with ‘problems or facts, but about the process of emergence of new relations which, potentially at least, can reconfigure what the very “fact” or “problem” might be’ (p. 175). He shows how speculative design enables ‘inventive problem making’, which ‘opens up a space for a reframing of the issues’ (Michael, 2012b: 539). He argues that speculative designers pursue the unexpected, expressive and creative, and that we should embrace this in our collaborations with them. So rather than social science ‘learning from’ art and design, he thinks we should talk of a ‘mutual “becoming with” of these disciplines, involving ‘artefacts that embody openness, ambiguity, playfulness’ (Michael, 2012a: 177–178).

Michael’s experiences resonate with ours on the Synthetic Aesthetics project, but a key difference is that our project engaged closely with critical design, and Michael draws a distinction between speculative and critical design. He thinks that a limitation of critical design is that it has a pre-defined target of critique. His concern is that ‘critique does not well accommodate the possibility of a co-emergence of researcher-and-researched’ since ‘the problem is pre-figured in critique, rather than inventively emergent’ (Michael, 2012a: 180, see Note 2). He maintains that since speculative designers do not set their work so directly against a particular sociotechnical future, the discussion can be more open-ended than is possible in critical design.

Questions arise, however, about whether we can (or whether practitioners do) distinguish speculative from critical design so strictly. Dunne and Raby (2013), the founders of critical design, call their book *Speculative Everything*, and they describe themselves as speculative designers (p. 100).^4^ They also distinguish their interest in ‘problem finding’ from the ‘problem solving’ of commercial design (Dunne and Raby, 2013: vii). ‘Problem finding’ seems very close to what Michael describes as ‘inventive problem making’.

We think the key issue is whether there is a difference between designs that have a specific target of critique and those that enable a more generative discussion. It could be argued that the Scatalog described above has a target of critique – the clean, shiny image of high-tech synthetic biology, which it makes mundane in a memorable manner. But as we have shown, it can be read in multiple ways by different actors. The cheese is even less directed against a specific target of critique. It challenges simplistic, ‘flattened’ electronic analogies that we see in synthetic biology, but it does far more than this in its ambivalent disgustingness and potential edibility.

In the Synthetic Aesthetics project overall, Daisy’s work (which she labels as critical design) exhibits the emergent characteristics that Michael sees in speculative design. We also find these characteristics in the work of the residents. Although Sascha, Sissel and Oron self-identified as artists rather than critical or speculative designers, the features of their work that they argued distinguished it as ‘art’ (its capacity to initiate new conversations, to be contested, to pose questions rather than deliver answers) are exactly those that we are identifying as features of an emergent form of critique. We think that how the work is labelled is less important than whether it provokes ongoing exploration and discussion.

Another way of thinking about the distinction between speculative and critical design is in terms of the collaborative nature of the development of the art/design artefact. While a critical designer’s work is often produced by a designer working alone, to be exhibited later in a museum or gallery, in what Michael calls speculative design the process does not rest so heavily on the designer bringing his or her expert skills to bear; it is more open-ended and the design object is coproduced with publics or users. A key feature of Synthetic Aesthetics, however, was that the work was essentially collaborative. The project did not support works of commentary produced by stand-alone artists or designers, but required the production of something that was jointly created with the paired scientist/engineer. If the relevant difference is an issue of expertise and whether it is asserted or shared, sharing was paramount in the Synthetic Aesthetics project. Because of their shared endeavours, we think the residents’ work exhibited the inventive problem making that Michael celebrates, as well as the creation of new objects and practices that is central to Barry et al.’s (2008) ‘logic of ontology’.

For example, without Hideo’s expertise in cyanobacteria and their circadian rhythms, the pair’s investigation of the temporal scope of these organisms would not have come about, and the poetic and humbling form that this project eventually took would not have transpired without the collaboration. Sascha and Sheref’s work was highly dependent on their initial discussions of the features of living things that are central to protocell research, but it is doubtful that either Sascha or Sheref would have come up with the idea of an evolving steam engine independently. Sissel and Christina’s production of human bacterial cheeses was dependent on the unlikely partnership of synthetic biology concepts and practices in scent design.

## Future directions

Inspired by the work of these pairs and our own interactions in the Synthetic Aesthetics project, we conclude by sketching the outlines of what we mean by an emergent form of critique – something that we hope to develop in future work.

We are calling this an ‘emergent’ form of critique is because it is necessarily co-produced. It is not the result of one group imposing their critical tools and perspectives, but is the outcome of a shared endeavour that brings people together from different disciplines. What is generated is thus more than (and different from) anything that could result from a single discipline. This relates to our discussion of ‘making’ above, because if what is important is what emerges from these shared collaborative endeavours, then it becomes less relevant who is a ‘maker’ and who is not.

In using the word ‘critique’, we are drawing on Latour (2010), who rejects the familiar notion of critique as ‘predicated on the discovery of a true world of realities lying behind a veil of appearances’ (pp. 474–475), and instead advocates a notion that is more about unexpectedness, openness and overspilling. It is ‘associated with *more*, not with *less*, with *multiplication*, not *subtraction*’ (Latour, 2004: 248, emphasis in original). Latour (2004) argues,

The critic is not the one who debunks, but the one who assembles. The critic is not the one who lifts the rugs from under the feet of the naive believers, but the one who offers the participants arenas in which to gather. (p. 246)

One way we want to explore and take forward this notion of critique is by attempting to create spaces where mutually transformative discussions can happen between STS, art/design and science/engineering, as well as other groups.

We think that an emergent form of critique requires a type of collaboration that is experimental, and that this could provide an ethos for interdisciplinarity more generally, beyond that of STS and art/design. Here we are building on work such as that by Marcus (2013), who aims to experiment with collaborative forms of anthropological knowledge production and raise new questions in the process, and Fitzgerald and Callard (2015), who work closely with neuroscientists, and argue that ‘novelty, serendipity and contingency might conjure a more constructive space of shared collaboration’ (p. 5). Such experimental collaborations are necessarily risky, because the outcomes will not be obvious from the outset, but will emerge from the process of collaboration itself.

If we are going to commit to being part of such experimental collaborations, we may also have to think of ourselves more explicitly as ‘participants’ rather than ‘spectators’ (Barad, 2007) in technoscientific worlds, because it is only if we participate that we can create something new together – whether this be knowledge, practices or things. We may have to admit our complicity and become part of the fields we study. We will lose distance, but we may gain something more unexpected.

What we take away from our involvement in the Synthetic Aesthetics project is that experimental collaborations with artists and designers can be playful, challenging, and sometimes transcendent. We might not always know exactly what we are doing, and we may have to walk forward without being sure that the ground is solid beneath our feet. But by working *with* artists and designers instead of making studies *of* them, we can together start to develop an emergent form of critique, which could allow novel discussions and explorations of sociotechnical complexity, and perhaps bring something new into being.

## Notes

1. One of the pairs, Oron Catts and Hideo Iwasaki, applied together.

2. http://www.echromi.com/ (accessed 18 October 2016).

3. http://syntheticaesthetics.org/residents/agapakis-tolaas (accessed 18 October 2016).

4. Under the heading of ‘conceptual design’, Dunne and Raby (2013) list speculative design, critical design, design fiction, design futures, antidesign, radical design, interrogative design, design for debate, adversarial design, discursive design, futurescaping and design art (p. 11).
